# Preventive effect of *Ligularia fischeri* on inhibition of nitric oxide in lipopolysaccharide-stimulated RAW 264.7 macrophages depending on cooking method

**DOI:** 10.1186/0717-6287-47-69

**Published:** 2014-12-15

**Authors:** Hee-Sook Park, Hye-Young Choi, Gun-Hee Kim

**Affiliations:** Plant Resources Research Institute, Duksung Women’s University, 419 Ssang mun-dong, Dobong-gu, Seoul, Republic of Korea

**Keywords:** Chlorogenic acid, Inflammation, *Ligularia fischeri*, Macrophage

## Abstract

**Background:**

*Ligularia fischeri* (common name Gomchwi) is known for its pharmaceutical properties and used in the treatment of jaundice, scarlet-fever, rheumatoidal arthritis, and hepatic diseases; however, little is known about its anti-inflammatory effect. In this study the influence of blanching and pan-frying on the anti-inflammatory activity of *Ligularia fischeri* (LF) was evaluated.

**Results:**

Fresh LF and cooked LF showed no significant effect on the viability of macrophages after 24 h incubation. Fresh LF was found to be the most potent inhibitor of nitric oxide (NO) production at 100 μg/ml, while pan-fried LF showed little inhibitory effect on lipoloysaccharide (LPS) stimulated murine machrophage RAW264.7 cells. In contrast with its effect on NO production, pan-fried LF showed significant attenuation of the expression of inducible nitiric oxide synthase (iNOS) compared with fresh LF. In the cooking method of LF, PGE_2_ production was not affected in the LPS-induced RAW 264.7 cells. In LPS-induced RAW 264.7 cells, pretreatment by fresh and cooked LF increased COX2 mRNA expression. The 3-O-caffeoylquinic acid content of blanching and pan-frying LF increased by 4.92 and 9.7 fold with blanching and pan-frying respectively in comparison with uncooked LF.

**Conclusions:**

Regardless of the cooking method, *Ligularia fischeri* exhibited potent inhibition of NO production through expression of iNOS in LPS-induced RAW264.7 cells.

## Background

*Ligularia fischeri* (common name *Gomchwi*) belongs to the family Compositae, which are perennial vegetable plants found mainly in damp shady regions besides brooks and sloping fields in Europe and Asia [1]. In Korea, *Gomchwi* is generally consumed as salted or fried after a blanching process and is then called *Chinamul*. The leaves of *L. fischeri* have been used for their pharmaceutical properties in the treatment of jaundice, scarlet-fever, rheumatoidal arthritis, and hepatic diseases [2]. Antioxidant activity of this plant has been demonstrated by several independent methods, indicating that the plant contains high amounts of antioxidant constituents [1, 3, 4]. In our previous study, *L. fischeri* exhibited a preventive myoglobin ratio against various reactive oxygen species (ROS) and reactive nitrogen species (RNS) [5]. In another study, *L. fischeri* leaf tea prepared by blanching fresh leaves in boiling water was recognized as a value-added functional food that contains biological constituents such as caffeoylquinic acid [6, 7]. Leaves of *L. fischeri* contain caffeoylquinic acid derivatives (CQA) as major phenolic constituents [7, 8]. Shang et al. [7] reported that a number of caffeoylquinic acid derivatives (CQAs) have been isolated and suggested that these represent the major phenolic constituents in the leaves of *L. fischeri.*

Inflammation, a central feature of many pathophysiological conditions, occurs in response to tissue injury and results in the development of various human diseases such as cancer and diabetes [9]. During an inflammatory response, macrophages regulate different intracellular signaling pathways and this result in the release of several inflammatory mediators such as cytokines [10]. These in turn induce pro-inflammatory enzymes including the inducible forms of nitric oxide synthase (iNOS) and cyclooxygenase (COX), which are responsible for increasing the levels of NO and prostaglandins (PGs) respectively [11]. Santos reported that plant natural polyphenols, namely caffeoylquinic acid derivatives, stimulated inflammatory mediator production (Dos Saontos et al. [12]). Lemongrass, which contained chlorogenic acid (3-caffeoylquinic acid), has anti-inflammatory activities via inhibition of cytokine expression [10]. Supplementation with anti-inflammatory materials is a possible preventive and therapeutic strategy for inflammation induced-diseases [13]. The objective of this study is to determine the therapeutic effects of *Ligularia fischeri* that has been subject to cooking processes involved in anti-inflammatory activities.

## Results

### Effect of cooked LF on cell viability

**Figure 1:**
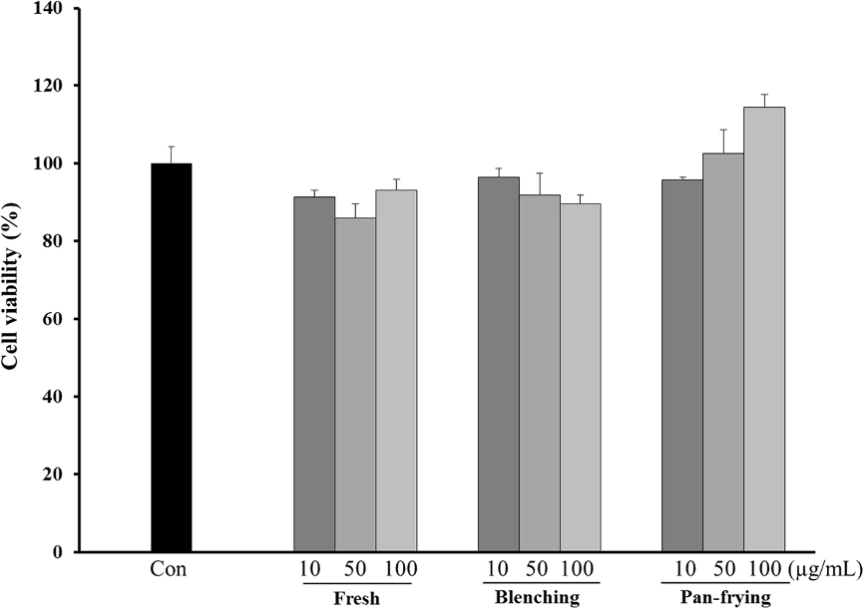
**Effect of LF on the viability of RAW264.7 cells.** Cultured cells were treated with different concentrations of uncooked and cooked LF for 24 hr. Data represent mean values of three experiments.

The cytotoxicity effects of LF on RAW264.7 cells were evaluated using MTT assay. RAW264.7 cells were incubated with differently cooked LF samples at various concentrations for an indicated cooking time. The result of the MTT assay showed that uncooked (fresh) and cooked LF, even at concentrations of 100 μg/mL, had no effect on cell viability in RAW264.7 cells, demonstrating that no effective cytotoxicity of LF was detected in any of the concentrations (Figure 1).

### Effects of cooked LF on LPS-induced NO production and expression of iNOS in RAW264.7 cells

**Figure 2:**
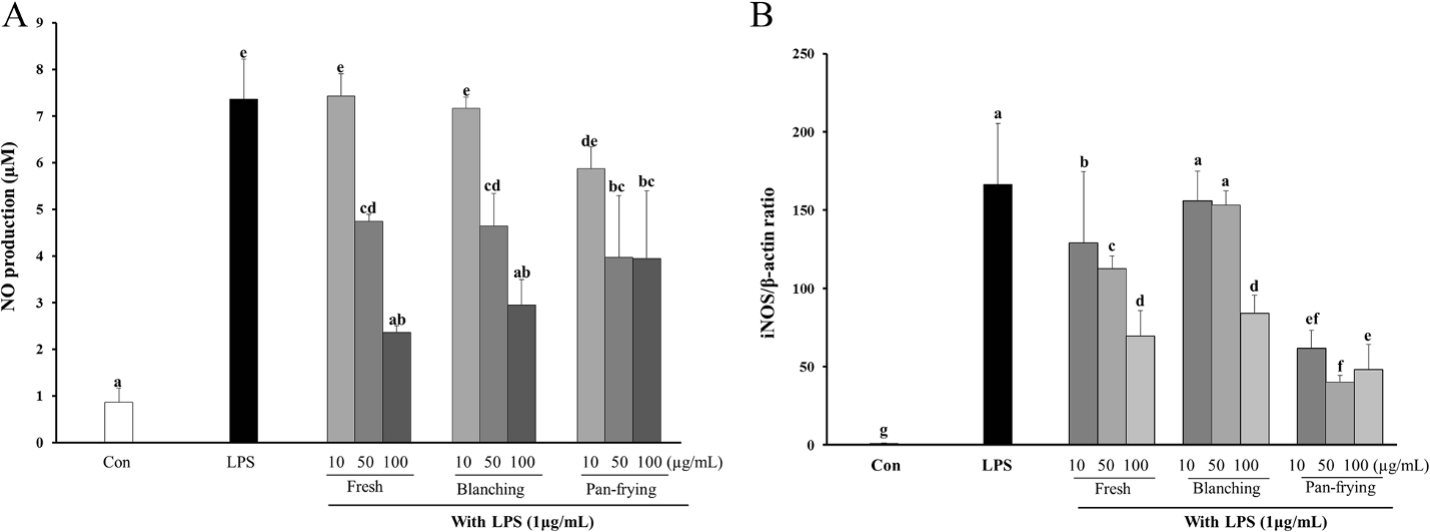
**Effect of LF on LPS-induced nitric oxide (NO) production and iNOS expression in RAW264.7 cells.** Cells cultured were pretreated with different concentrations of LF for 1 hr and stimulated with 1μg/mL LPS for 24 hr. Then **(A)** nitrite accumulation was assessed as a measure of NO production, and **(B)** Real-time PCR analysis was used to examine iNOS expression. Groups with different letters are significantly different from each other, *P* < 0.05.

The preventive effect of cooked LF on NO (Nitrite) production was evaluated after induction of inflammation. NO accumulation was determined in cell culture media stimulated with LPS in the presence or absence of cooked LF. NO production in LPS-stimulated cells treated with all of the differently cooked LF showed significant inhibition in a dose-dependent manner (Figure 2A). Pan-fried LF showed greater inhibition of NO production than other cooking methods at low concentrations (10 μg/mL). At the high dose (100 μg/mL), both uncooked and blanched LF showed significant inhibition of NO production in LPS-induced RAW264.7 cells. Based on these results, we examined whether or not LF affected the expression level of iNOS. Expression levels of iNOS mRNA increased markedly after treatment with LPS for 24 hr compared with the control group; however, cells pretreated with LF showed inhibition of iNOS expression levels following LPS stimulation (Figure 2B). Uncooked LF (fresh) significantly inhibited mRNA expression levels of iNOS in LPS-induced RAW264.7 cells at all of the tested concentrations. Likewise, pan-fried LF showed significant inhibition of mRNA expression of iNOS at all the tested concentrations, while blanched LF pretreatment attenuated mRNA expression levels of iNOS in a significant manner at the high dose (100 μg/mL).

### Effects of cooked LF on LPS-induced PGE_2_ production and expression of COX2 in RAW264.7 cells

**Figure 3:**
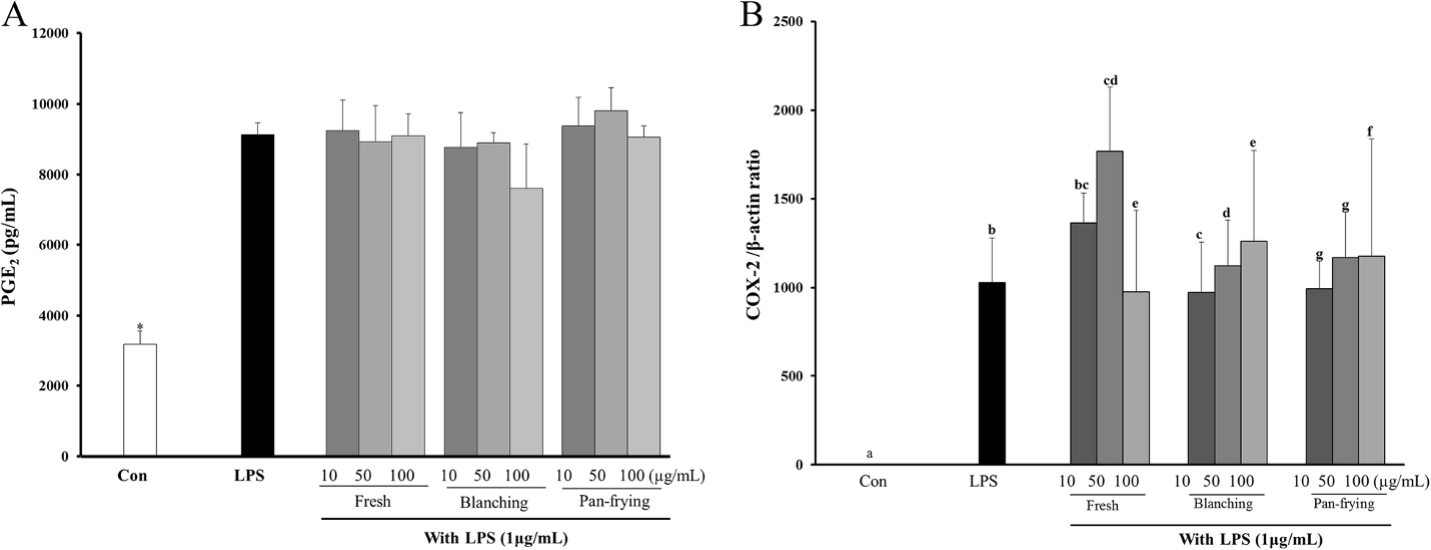
**Effect of LF on LPS-induced prostaglandin E**_**2**_ **(PGE**_**2**_ **) production and COX2 expression in RAW264.7 cells.** Cells cultured were pretreated with different concentrations of LF for 1 hr and stimulated with 1μg/mL LPS for 24 hr. Then **(A)** PGE_2_ production was measured in the culture medium, and **(B)** Real-time PCR analysis was used to examine iNOS expression. Groups with different letters are significantly different from each other, *P* < 0.05.

COX2 levels were examined using PGE_2_ immunoassay to determine whether or not LF-mediated inhibition was related to the modulation of PGE_2_ release. Although PGE_2_ production was not affected in the LPS-induced RAW 264.7 cells (Figure 3), pretreatment by LF showed changes in COX2 mRNA (Figure 3B). COX2 expression increased markedly on treatment with LPS and was significantly inhibited by uncooked (fresh) LF at the high dose (100 μg/mL). Also the LPS-induced COX2 increase was affected by blanched LF, while pan-fried LF showed a significant increase in levels of COX2 expression at all treated doses.

### Effect of chlarogenic acid as a bioactive component from LF on inflammatory responses

HPLC was used for the identification and quantification of 3-O-caffeoylquinic acid, as a bioactive component of LF. 3-O-caffeoylquinic acid was identified according to relative retention time as of the standard. All of the cooking methods exhibited greater amounts of 3-O-caffeoylquinic acid compared with the uncooked LF (fresh) with increases of 4.92 and 9.7 fold for blanching and pan-frying (Table 1).

**Table 1:** **3-O-caffeoylquinic acid content of** ***Ligularia fischeri***

Cooking method	Content (μg/100 μg/mL of ext.)	Change ratio (fold) (over uncooked LF)
Uncooked (fresh)	0.647	-
Blanching	3.187	4.92
Pan-frying	6.276	4.92

## Discussion

For vegetables, cooking (boiling, microwaving, pressure-cooking, grilling, baking, and frying) can have a profound effect on both the cell walls and nutritional value [14, 15]. Cooking processes bring about a number of changes in the chemical composition of vegetables [16]. In this study, when *L. fischeri* was submitted to blanch and pan-fry variations appeared in the concentration of 3-O-caffeoylquinic acid (Table 1). It was observed that the lower the initial 3-O-caffeoylquinic acid content, the higher the increase caused by the cooking treatment. The concentration of phenolic acids is highest in the outer layers of some vegetables and these areas are exposed to water [17]. Although total phenolics are usually stored in vegetables in pectin or cellulose networks and can be released during thermal processing, individual phenolic compounds may sometimes increase because heat can break the supramolecular structure [18]. Considering the above, the cooking process could have had a significant influence on the concentration of 3-O-caffeoylquinic acid through cell tissue distribution in *L.fischeri*.

Many studies have reported the isolation of bioactive components from extracts of *L. fischeri* and have evaluated their antioxidant activities [2, 7, 19, 20]. In our previous study, the antioxidant activities of extracts of LF were changed by cooking processes [5]. Lee and Choi reported that LF showed anti-inflammatory activities using carrageenan in formalin-induced experimental animal models [21]. Also LF modulated the inflammatory process by suppressing various genes in human synovial cells [22]. In this study, blanched LF showed greater inhibition of NO production in LPS-induced RAW264.7 cells compared with uncooked and pan-fried LF (Figure 2A). As a bioactive component of LF, 3-O-caffeoylquinic acid significantly inhibited NO production and iNOS and COX2 expression in the 0 ~ 20 μM range [23]. In this study, although LF did not cause any decline of COX2 expression, it inhibited NO production and iNOS expression. These results might anti-inflammatory effects of LF were affected through COX2-independent signaling in LPS-induced macrophage.

## Conclusions

Regardless of the cooking method, *L. fischeri* exhibited potent inhibition of NO production through expression of iNOS in LPS-induced RAW264.7 cells. This indicates that the anti-inflammatory effects of LF were not only caused by the 3-O-caffeoylquinic acid content in LF and that, after going through the cooking process, LF may influence the anti-inflammatory response. Based on these results, *L. fischeri* may be beneficial for the prevention of anti-inflammatory diseases.

## Methods

### Materials

*Ligularia fischeri* (LF) was collected at Inje-gun, Gangwon-do, Korea. RAW264.7 cells were obtained from the Korean Cell Line Bank (Seoul, Korea). Low glucose (1000 mg/ml) Dulbeco’s modified Eagle’s medium (DMEM), fetal bovine serum (FBS), and penicillin-streptomycin cocktail were purchased from WELLGENE (Daegu, Korea). Lipopolysaccharide (LPS, Escherichia coli O55:B5), 3-[4, 5-dimethylthiazol-2-yl]-2, 5-diphenyl-tetrazolium bromide (MTT), and 3-O-caffeoylquinic acid were purchased from Sigma-Aldrich (St. Louis, MO, USA). Griess reagent was obtained from Promega (Madison, WI, USA). Nitric oxide (NO) and prostaglandin E_2_ (PGE_2_) assay kit were purchased from R&D System (Minnesota, USA). Reverse transcription and polymerase chain reaction premixes were from Applied Biosystems (Carlsbad, CA, USA).

### Sample preparation

*L. fischeri* was collected at Inje-gun, Gangwon-do, Korea. LF was cooked in our laboratory, after cleaning and washing with water and cutting into small pieces. The LF was divided to provide 3 samples for fresh, blanching and pan-frying the rest was subjected to different cooking methods.

1. Blanching in a stainless steel vessel: Washed LF (200 g) was added to water (2L) and blanched for 3 min.
2. Pan-frying: Washed LF (200 g) was placed in a frying pan with oil and stirred for 3 min.

LF extracts were obtained using 70% ethanol with sonication (POWERSONIC 420, 700 W, 50/60 Hz, Hwanshin Tech.) for 40 min twice. Then the extracts of LF were filtered, evaporated (ELISA EVAPORATOR NVC-2000, SB-1000, DPE-1210, CA-1112, ELISA, Japan), and freeze-dried (FD5510, IlShin Lab Co., Ltd., Korea) to make powder samples. All samples were diluted to a 10 mg/mL concentration and used for the anti-inflammatory sample.

### HPLC analysis

Reverse-phase high performance liquid chromatography (HPLC) was conducted using a Dianex u-300 system (Milford, MA, USA) that consists of Ultimate 3000 pumps, autosampler, and UV detector. The Chromeleon chromatographic system was employed to analyze the HPLC data. Chromatographic separation was accomplished using an Atlantis dC_18_ reverse phase column (Waters, 4.6 × 150 mm, 5 μm) and the elution was monitored at 300 nm. For separation, solvents A (Acetonitriles, ACN) and B (0.02% aqueous phosphoric acid, v/v) were used. The gradient program used was as follows: initial 0-6min, linear change from A-B (13:87, v/v) to A-B (15: 85, v/v); then held for 3 min; 9–17 min, linear change from A-B (15:85, v/v) to A-B (19:81, v/v); 17–28 min, linear change from A-B (17: 83, v/v) to A-B (28: 72, v/v); and then held for 9 min. The flow rate was 0.6 mL/min and an aliquot of 10 μL was injected.

### Cell culture

Murine RAW264.7 macrophages were cultured in DMEM medium containing 10% FBS, peniciln, and streptomycin in a 5% CO_2_ humidified incubator at 37°C. RAW 264.7 cells were grown in 48-well plates at a density of approximately 5 × 10^4^ cells per well.

### Cell viability

Cells were treated with different concentrations (10, 50, 100 μg/mL) of LF for 24 hr. After that, the cells were incubated with MTT reagent, which was added to the culture medium at a final concentration of 0.5 mg/mL, for 4 hr in a 5% CO_2_ humidified incubator at 37°C. The resultant dark blue crystals were dissolved using dimethyl sulfoxide (DMSO) and absorbance values were measured at 540 nm.

### Measurement of nitric oxide (NO) production

The production of NO was determined by measuring the accumulated level of nitrite, an indicator of NO in the supernatant. The RAW 264.7 cells were pretreated with or without LF for 1 hr. After LPS (1 μg/mL) was added to the cultured medium for 24 hr, nitrate levels were measured in cell culture supernatants according to the Griess reaction (1% sulfanilamide, 0.1% *N*-[naphthyl] ethylenediamine dihydrochloride, and 5% phosphoric acid) at room temperature for 10 min. Absorbance of the mixture at 550 nm was measured in a micro-plate reader (SpectraMax M2, Molecular Devices, USA). Nitrate concentration was calculated by comparison with a nitrite standard curve.

### Measurement of prostaglandin E_2_ (PGE_2_) production

RAW 264.7 cells were seeded in 48-well plates (5 × 10^4^ cells per wells) and incubated for 24 hr. The cells were treated with LF or a vehicle in the presence of LPS (1 μg/μL) for an additional 24 hr. PGE_2_ production in the cell supernatant was evaluated by PGE_2_ Parameter Assay kit following the manufacturer’s instructions.

### Real-time quantitative polymerase chain reaction (RT-PCR)

RAW 264.7 cells were cultured in 6-well plates (5 × 10^5^ cells per well) for 24 hr. The cells were treated with LF or a vehicle in the presence of LPS (1 μg/μL) for an additional 24 hr. Total RNA was isolated from the cells using RNAeasy kit (Qiagen) and then total RNA reverse-transcribed using a High Capacity RNA-to-cDNA™ Kit (Applied Biosystems) in order to produce cDNAs. Quantitative RT-PCR was performed in a Power SYBR® Green Master Mix (Applied Biosystems) under a STEPONE PLUS (Applied Biosystems), and the results were analyzed with the Stepone Software VER. 2.1 supplied with the machine. The housekeeping gene β-actin was used as an internal standard to quantify the levels of iNOS and COX2 mRNA. Parameters of RT-PCR reaction were 95°C for 5 min for one cycle, then 95°C for 15 sec, 61°C for 30 sec, and 72°C for 30 sec for 40 cycles. The fluorescence signal was detected at the end of each cycle. The primers used in the experiment were iNOS, forward: 5'- CCC TTC CGA AGT TTC TGG CAG CAG C -3', reverse: 5'- GGC TGT CAG AGC CTC GTG GCT TTG G -3'; COX2, forward: 5'- TCT CCA ACC TCT CCT ACT AC -3', reverse: 5'- GCA CGT AGT CTT CGA TCA CT -3'; and β-actin, forward: 5'- CCG TCT TCC CCT CCA TCG T -3', reverse: 5'- ATC GTC CCA GTT GGT TAC AAT GC -3'.

### Statistical analysis

All experiments were repeated three times. All data are expressed as mean values standard deviation (SD). Statistical evaluations were made by ANOVA followed a Tukey’s HSD multiple comparison test. A value of *p* < 0.05 was considered significant.
